# Automated vortex-assisted-liquid-liquid microextraction with injector-based derivatization for GC-MS/MS analysis of 1,3-dichloropropan-2-ol and 3-chloropropane-1,2-diol in food contact paper products

**DOI:** 10.1007/s00216-025-06133-2

**Published:** 2025-10-21

**Authors:** Malte Hübschen, Fabrian Brenz, Torsten C. Schmidt

**Affiliations:** 1 Faculty of Chemistry, Instrumental Analytical Chemistry, University of Duisburg-Essen, Universitätsstraße 5, 45141 Essen, Germany; 2 Axel Semrau GmbH, Stefansbecke 42, 45549 Sprockhövel, Germany; 3 CVUA-MEL, Joseph-König-Straße 40, 48147 Münster, Germany

**Keywords:** 3-MCPD, 1,3-DCP, Food contact materials, Injector-based derivatization, VALLME, Automated sample preparation

## Abstract

The analysis of 1,3-dichloropropan-2-ol (1,3-DCP) and 3-chloropropane-1,2-diol (3-MCPD) in paper products and cardboard boxes used as food contact materials is of great importance due to the potential health risks associated with the intake of these contaminants. Both compounds, which originate from wet strength agents used in paper production, pose significant health concerns: 1,3-DCP and 3-MCPD are classified as possibly carcinogenic to humans and suspected to be carcinogenic in humans, respectively. This study presents a novel, automated analytical method for the analysis of a cold water extract—a widely used procedure for isolating these analytes from paper products and cardboard boxes. Method development included the implementation of vortex-assisted liquid-liquid microextraction (VALLME) with a focus on reducing solvent consumption and improving environmental sustainability and the systematic investigation of derivatization techniques. Optimization of the extraction solvent, extraction time, and derivatization parameters using vial-based and injector-based approaches resulted in a lean and rapid method with low limits of detection (0.01 µg/L for 1,3-DCP and 0.16 µg/L for 3-MCPD). The method showed good precision with intra-day repeatabilities of 3.6–3.7% for 1,3-DCP and 3.6–5.9% for 3-MCPD and inter-day repeatabilities of 2.6–3.6% and 4.0–7.6%, respectively. Recoveries ranged from 100 to 102% for 1,3-DCP and from 90 to 99% for 3-MCPD. Comparative assessment with a reference method confirmed good precision and comparability of the method. Improved environmental performance was quantified by the analytical greenness metric for sample preparation.

**Graphical abstract:**

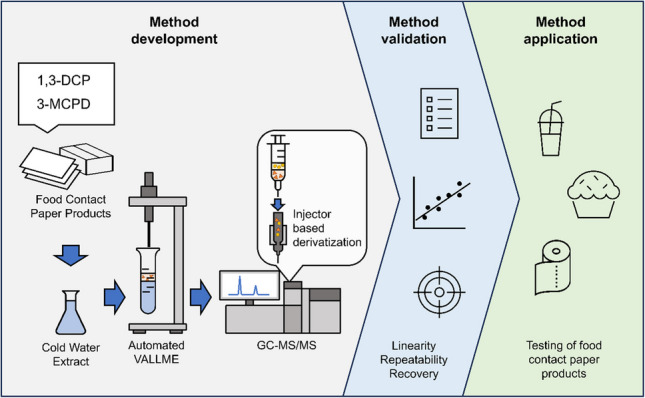

**Supplementary Information:**

The online version contains supplementary material available at 10.1007/s00216-025-06133-2.

## Introduction

Paper, a versatile technical product, serves numerous purposes and undergoes modification with specific additives tailored to its intended applications [1]. Cardboard, for instance, represents a specialized form of stabilized paper primarily employed in packaging technology [2]. Among the potential paper additives, polyamidoamine-epichlorohydrin (PAE) resin stands out as a wet strength agent, crucial for preventing paper fiber separation in moist conditions. In the production of polyamidoamine epichlorohydrin (PAE) resins, 1,3-dichloropropan-2-ol (1,3-DCP) is mainly formed when epichlorohydrin reacts with chloride ions during the final stages of resin synthesis, while 3-chloropropane-1,2-diol (3-MCPD) is formed by the hydrolysis of epichlorohydrin or the PAE resin intermediates [3–5]. These chloropropanols have been detected across various food contact materials, including coffee filters, milk cartons, drinking straws, kitchen rolls, muffin cups, and bagasse plates and bowls [6–9].

In studies in rats, 3-monochloropropane-1,2-diol (3-MCPD) has been shown to cause severe renal toxicity and the development of benign tumors, including those of the kidney, Leydig cells, and mammary glands [10]. 3-MCPD is classified as suspected to be carcinogenic in humans [11]. Despite some positive genotoxicity tests in vitro, there is no evidence that 3-MCPD is genotoxic in vivo in any organ tested [11]. The European Food Safety Authority (EFSA) has established a tolerable daily intake (TDI) for 3-MCPD of 2 µg/kg body weight per day [12]. In addition, 3-MCPD has been found to be hepatotoxic in rats, with repeated oral administration leading to tissue changes and increased liver weight [13]. Long-term exposure of rats to 1,3-DCP resulted in the development of tumors in several organs. This compound has been classified as both genotoxic and carcinogenic [14]. It is therefore classified as a category 1B carcinogen [15]. The German Federal Institute for Risk Assessment (BfR) has recommended limits for these contaminants in water extracts from paper and board to ensure consumer safety [16]: No 1,3-DCP must be detectable in the cold water extract of the product, with a detection limit of 2 μg/L. Additionally, the transfer of 3-MCPD into the cold water extract should be minimized, aiming to keep it as low as possible. A guideline value of 12 μg/L should not be exceeded. For compliance testing, it is therefore necessary to establish a limit of quantification (LOQ) of 1 µg/L for these substances.

The official German test method B 80.56-2 mandates the preparation of a cold water extract for analyzing 3-MCPD and 1,3-DCP [17]. Post-extraction, analytes undergo enrichment with diatomaceous earth through column chromatography, followed by elution and derivatization with *N*-heptafluorobutyrylimidazole (HFBI). Subsequently, gas chromatography (GC) coupled with an electron capture detector (ECD) is employed for analysis. However, the ECD analysis faces challenges due to the unspecific nature resulting from various matrix components in cold water extracts, rendering it outdated [7]. Recent methodologies utilize mass spectrometry instead, enhancing selectivity [7, 8, 18]. Furthermore, newer derivatization reagents, replacing HFBI, mitigate background noise issues, contributing to improved analysis [19]. Silylating agents have emerged as a favorable alternative in recent methods, alongside mass spectrometric detection, resulting in significantly enhanced selectivity [7, 8, 18, 19].

The preparation of a cold water extract followed by column chromatography with diatomaceous earth as described in the official method B 80.56-2 corresponds to the methods currently used in official and commercial laboratories. However, in contrast to B 80.56-2, the derivatization is often performed with trimethylsilyl-*N*-methyltrifluoroacetamide (MSTFA) and a mass spectrometer is used [7, 8]. This approach is employed as the reference method for comparison in the study.

The reference method had several limitations that necessitated the development of a new sample preparation protocol [8]. In particular, all sample preparation steps were performed manually, resulting in increased labor and potential variability. In addition, the reference method required significant amounts of organic solvents and derivatization reagents, raising concerns about environmental impact and cost-effectiveness.

The primary aim of this study was to develop a novel, automated method for analyzing 1,3-DCP and 3-MCPD, aiming for a LOQ of 1 µg/L to meet compliance testing requirements. In order to achieve this objective, various derivatization techniques and the application of vortex-assisted liquid-liquid microextraction (VALLME) were investigated. VALLME is an extraction technique that has been shown to rapidly achieve phase equilibrium with minimal solvent consumption [20]. To assess the environmental responsibility of the newly developed method, its environmental friendliness was evaluated using the Analytical GREEnness Metric for Sample Preparation (AGREEprep) tool [21].

## Experimental section

### Reagents and materials

The standards 1,3-dichloropropan-2-ol (1,3-DCP), d5-1,3-dichloropropan-2-ol (d5-1,3-DCP), 3-chloropropane-1,2-diol (3-MCPD), and d5-3-chloropropane-1,2-diol (d5-3-MCPD) were obtained from Toronto Research Chemicals (Canada). For the preparation of the mixed spiking standard, 1,3-DCP and 3-MCPD were dissolved and diluted to 0.1 mg/L and 1 mg/L, respectively, in ultrapure water (VWR, USA). For the internal standard, d5-1,3-DCP and d5-3-MCPD were dissolved in ultrapure water and diluted to a final concentration of 0.1 mg/L. For method development, d5-1,3-DCP and d5-3-MCPD were dissolved in methyl tert-butyl ether (MTBE), diethyl ether (Et_2_O) and ethyl acetate (EtOAc), then diluted to 0.1 mg/L. MTBE, Et_2_O, and EtOAc were obtained from Sigma-Aldrich (Germany). Sodium chloride was purchased from Sigma-Aldrich (Germany). *N*-Methyl-*N*-(trimethylsilyl)trifluoroacetamide (MSTFA) was ordered from Macherey–Nagel GmbH & Co. KG (Germany). The supplier of propylene carbonate was Sigma-Aldrich (Germany). The 2-mL and 20-mL vials with the corresponding magnetic caps came from VWR (USA). The 10-mL bulb pipette class AS was ordered from Carl Roth GmbH + Co. KG (Germany).

To demonstrate the applicability of the introduced approach, various paper-based products were analyzed, including drinking straws, kitchen rolls, and cardboard bowls. These samples were sourced from inter-laboratory comparison studies and retail outlets to represent commonly used consumer products.

### Instrumentation

#### Gas chromatography-tandem mass spectrometry

An EVOQ TQ MS 456-GC (Bruker, USA) was used for chromatographic separation and detection. The GC-MS/MS was equipped with a split/splitless (SSL) injector (Bruker, USA) and a programmed temperature vaporization (PTV) injector inlet with fritted liner (OPTIC-4 multimode injector, GL Sciences, Netherlands). For the purpose of SSL injections, the injector was maintained at a temperature of 250 °C unless otherwise indicated. The splitless sampling time was set to 60 s, after which a flush of 60 s was initiated with a split of 100, followed by a split of 10. In the case of PTV injections, the injector was initially set to 60 °C for a duration of 5 s, followed by a gradual increase at a rate of 2 °C/s to a final temperature of 250 °C, which was maintained until the conclusion of the GC run. The injector operated in splitless mode for a period of 90 s, after which it was flushed with a split of 100 for a duration of 1 min, followed by a split of 10. However, exceptions were made in the optimizations where the heating rate and splitless time were varied. A deactivated metal retention gap (2 m × 0.53 mm) sourced from Trajan (Germany) was incorporated as a precolumn. This addition was for handling the high boiling keeper, ensuring efficient separation and analysis. An Rtx-5MS column (30 m × 0.25 mm × 0.25 µm) with a 5% phenyl-95% methyl polysiloxane phase (Restek, Germany) was used for the analytical separation. Helium 5.0 (Air Products, Germany) at a flow of 1.5 mL/min was used as the mobile phase. The column temperature program started at 60 °C for 2 min, increased at 20 °C/min to 90 °C, 10 °C/min to 150 °C, and 50 °C/min to 250 °C, held for 4 min. The transfer line temperature was set at 230 °C and the EI ion source temperature at 200 °C. The analytes were measured in multiple reaction monitoring (MRM) mode. The mass transitions of the derivatized analytes with the corresponding collision energies (CE) can be found in Table 1.

**Table 1:** Mass transitions of the derivatized analytes of the multiple reaction monitoring measurement with the corresponding collision energies (CE)

Compound	Quantifier		Qualifier 1		Qualifier 2	
	m/z	CE [V]	m/z	CE [V]	m/z	CE [V]
1,3-DCP-TMS	185 > 93	5	151 > 73	5	187 > 93	9
*d*_5_−1,3-DCP-TMS	190 > 93	5	154 > 73	5	192 > 93	9
3-MCPD-2TMS	239 > 147	3	116 > 101	5	241 > 147	9
*d*_5_−3-MCPD-2TMS	244 > 147	3	119 > 104	5	246 > 147	9

#### Autosampler

**Fig. 1:**
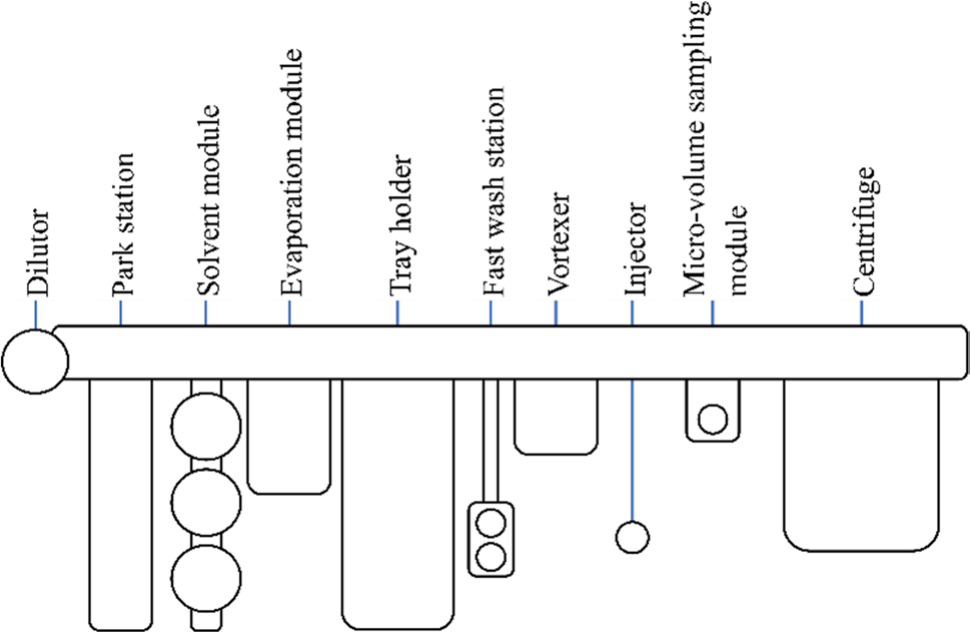
Schematic structure of the robotic tool change (RTC) autosampler with modules for automated vortex-assisted liquid-liquid microextraction (VALLME)

In this study, a robotic tool-changing autosampler (RTC PAL3, CTC Analytics, Switzerland) was configured with several specialized modules to automate vortex-assisted liquid-liquid microextraction (VALLME) (Fig. 1).

- Dilutor tool: This tool contained a syringe pump that delivered ethyl acetate, the extraction solvent for the VALLME process, to the sample.
- Park station: The park station contained three tools:
  - DIL tool: This tool was connected to the syringe pump and was used for liquid handling tasks.
  - Two liquid tools: These tools were equipped with syringes of 1 mL and 10 µL capacity, respectively.
- Solvent module: This module stored the internal standard and keeper.
- Tray holder: The tray holder was configured to hold both 20-mL and 2-mL sample vials.
- Fast wash station: This station was equipped with a solvent reservoir and cleaned the syringes between operations to prevent cross-contamination.
- Micro-volume sampling module: The micro-volume sampling module is designed to aspirate liquid from small volumes of liquid in flat-bottomed vials, and inject it into the measurement system (see “Micro-volume sampling module”).

### Automated sample preparation

The cold water extracts of paper products investigated in this study were extracted according to the standardized method B 80.56-2 of the Federal Office of Consumer Protection and Food Safety of Germany [17].

Using a 10-mL bulb pipette, 10.0 mL of the cold water extract is manually transferred to a 20-mL vial. Sodium chloride is added to give a final mass of 3.7 g. The 20-mL vial is sealed with a magnetic screw cap and placed on the autosampler.

After manual addition of NaCl and vial sealing, all subsequent steps were executed automatically by the PAL-based autosampler. The autosampler added 100 µL of internal standard solution to the sample, vortexed, and added 1.2 mL of ethyl acetate as extraction solvent. For extraction, the sample was vortexed at 2000 rpm for 5 min. The sample was centrifuged at 4800 rpm for 2 min. One milliliter of the supernatant was transferred to a 2-mL vial along with 20 µL of propylene carbonate as a polar, aprotic keeper. Using an evaporation module, ethyl acetate was removed under vacuum at 50 °C, 450 rpm, and 200 mbar for 5 min. The residue was diluted with 30 µL ethyl acetate and vortexed for 5 s at 2000 rpm. For injection, the vial was placed in a special micro-volume sampling module. The injection syringe first aspirated 1 µL of MSTFA, then 0.2 µL air buffer, and then 1 µL of the extracted sample. Derivatization was performed in the PTV injector during injection using the PTV injection settings described in the “Gas chromatography-tandem mass spectrometry” section. For the instrument methods for the autosampler, GC-MS/MS and PTV methods, view the electronic supplementary material S1.

### Statistical analysis

For the statistical analysis, Excel for Microsoft 365 and SPSS version 29 were employed. Significant differences between extraction solvents were assessed using ANOVA (*α = 0.05*) and Tukey’s test (*α = 0.05*). ANOVA test (*α = 0.05*) was utilized to determine significant differences in extraction times. Evaluation of OFAT for derivatization in the Agitator and DOE optimization for derivatization in the injector was conducted using Design-Expert version 22, employing response surface and numerical analysis. Calibration was examined for linear regression using the Mandel test with Excel for Microsoft 365.

## Results and discussion

### Method development

#### Selection of organic solvent for extraction

An initial extraction protocol with a 15-min vortex time was used to approach equilibrium between aqueous and organic phases while screening candidate solvents for VALLME. For solvent selection, a blank cold water extract (10.0 mL) was spiked to 10 µg/L of each analyte and salted with NaCl to a final mass of 3.5 g to promote phase separation and salting-out. To each spiked extract, 1.2 mL of the candidate extraction solvent (ethyl acetate, diethyl ether or methyl tert-butyl ether) was added, and the mixture vortexed automatically at 2000 rpm for 15 min. After centrifugation (4800 rpm, 2 min), 1.0 mL of the organic phase was transferred into a 2-mL vial containing 20 µL propylene carbonate as keeper and 100 µL of the internal standard solution (d5-labelled analogs dissolved in the same solvent as used for extraction). The extract was evaporated under vacuum (50 °C, 450 rpm, 200 mbar) for 5 min, the residue redissolved in 20 µL ethyl acetate, and the aliquot processed for derivatization. For vial-based derivatization, a scaled-down MSTFA protocol adapted from Korte et al. [8] was followed (10 µL MSTFA; 60 °C; 30 min; 450 rpm). Analyte/internal standard area ratios were compared to a solvent-spiked control (10 µg/L) to determine extraction performance.

**Fig. 2:**
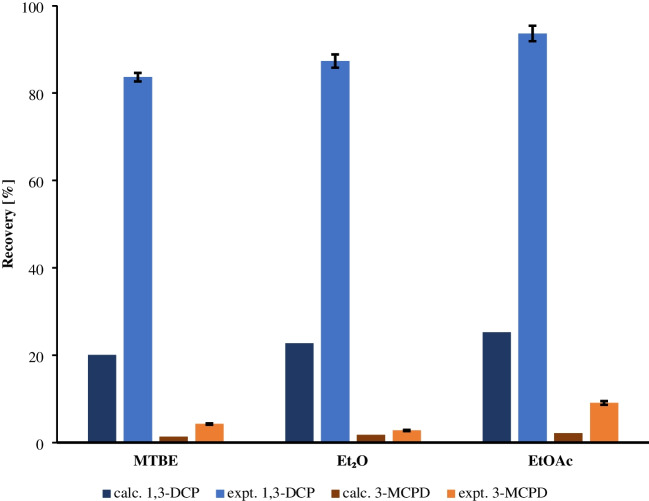
Extraction efficiency for 1,3-dichloropropan-2-ol (1,3-DCP) and 3-chloropropane-1,2-diol (3-MCPD) with methyl tert-butyl ether (MTBE), diethyl ether (Et_2_O) and ethyl acetate (EtOAc) at an analyte concentration of 10 µg/L from fivefold determinations. The experimental (expt.) recoveries refer to the sodium chloride saturated water and solvent mixture described in this study. The error bars indicate the relative standard deviation of the fivefold determination. The calculated (calc.) recoveries were determined using the partition coefficients from the UFZ-LSER database and refer to a water-solvent mixture [22]

Experimental extraction efficiencies were evaluated by fivefold determinations and contrasted with theoretical partitioning predicted from the UFZ-LSER database [22]. Excess NaCl increased the measured partitioning relative to the theoretical estimates (experimental efficiencies were on average ≈3.5 × higher than those calculated from the UFZ-LSER data; Fig. 2, Tables S1 and S2), demonstrating a salting-out effect [23]. The different polarities of the analytes governed the observed behavior: 1,3-DCP (one hydroxyl) partitioned substantially into the organic phase, whereas 3-MCPD (two hydroxyls) remained predominantly in the aqueous phase. Ethyl acetate provided the best result: an extraction efficiency of 88% for 1,3-DCP and 9% for 3-MCPD. One-way ANOVA followed by Tukey’s test (*α* = 0.05) confirmed that the solvent differences were statistically significant.

Low extraction yield of highly polar, dihydroxyl analytes in microextraction formats is a well-known phenomenon and has been reported for similar microextraction approaches; it is principally driven by analyte polarity and hydrogen-bonding capacity, and salting-out can only partially offset these intrinsic partitioning limitations [24–28]. Importantly, this study’s protocol uses isotope dilution (d5-1,3-DCP and d5-3-MCPD) with the internal standard added before extraction and derivatization. Stable isotope dilution is an established strategy in trace GC-MS to correct for differential losses occurring during sample preparation and derivatization; therefore, isotope correction compensates for the low extraction efficiency [29, 30].

In support of this approach, the method validation—including blind matrix spike experiments—produced consistent isotope-dilution corrected recoveries and precision despite the low yield for 3-MCPD (see “Analytical performance characteristics,” Table 2). These results demonstrate that the isotope-corrected method fulfills the required analytical performance (precision, LOQ) for the intended compliance testing.

Given the study’s goals of full automation, minimized solvent consumption, and high throughput, a single 1.2-mL VALLME step using ethyl acetate was selected. This approach keeps the workflow compact and easily automatable while minimizing solvent and reagent consumption, which is an important consideration under green analytical chemistry principles. It also enables higher sample throughput while achieving the method LOQ after isotope correction. The chosen solvent volume (1.2 mL) is smaller than that used in conventional extraction methods and falls within the range reported for microextraction approaches [31]. Ethyl acetate was also preferred because of its favorable toxicological and environmental profile compared to more hazardous solvents.

However, if a laboratory adopting this method requires higher uncorrected extraction recovery as a formal quality criterion, it can be adapted. Multistep extraction (e.g., repeated VALLME cycles), DLLME, and µSPE have all been reported to increase raw recovery for polar analytes [32]. Nevertheless, these alternatives require additional time, solvents, or dispersants; greater handling complexity; and additional hardware or cartridge consumables. For instance, DLLME can cause significant enrichment but typically requires dispersants and careful handling during phase separation [33]. µSPE can deliver high yields for polar analytes in automated setups but requires disposable cartridges, producing more waste [34]. Therefore, single-step VALLME has been retained as a validated, lean option, and it should be noted that multistep protocols or µSPE represent straightforward adaptation pathways when a laboratory’s quality policy mandates higher raw extraction yields.

#### Extraction time

After solvent selection, extraction kinetics were examined to define a minimal yet robust vortex time compatible with automation. A blank cold water extract was spiked at 1 µg/L and 50 µg/L. Vortex time was varied (1, 2, 3, 5, 7, 10 min) at 2000 rpm, and each condition was measured in quintuplicate to assess both mean recovery and variability. For the extraction efficiency screening and time-course experiments only, isotopically labeled internal standards were added after phase separation to avoid compensating for extraction losses; therefore, the recoveries in Fig. S1 and Table S3 represent raw extraction yields. For all quantitative measurements (calibration, validation, samples), internal standards were added before extraction and derivatization to enable full isotope-dilution correction.

The data showed only a marginal increase in extracted analyte with longer vortex times; one-way ANOVA (*α* = 0.05) indicated no statistically significant differences in mean recovery across the studied times at either concentration. For 1,3-DCP, precision at 50 µg/L was consistently good across times (RSD 1.7–4.6%), while at 1 µg/L RSDs were higher at short times (e.g., 25% at 1 min) but improved at 5 min (≈ 8%). For 3-MCPD, the 1 µg/L series showed larger RSDs (27–47%) across times, consistent with its low raw extraction in single-step VALLME and operation near the LOQ; at 50 µg/L, precision was acceptable and improved with mixing (RSD 8.8% at 1 min decreasing to ~ 2.5-5% for ≥ 5 min). Balancing precision with autosampler logistics (syringe washes and inter-step handling), 5 min was adopted. Importantly, the calibration (1–150 µg/L) and all validation measurements were performed under this fixed condition with isotope-dilution, for which the calibration was linear by Mandel’s test and matrix-matched precision at 1 µg/L met targets (Table 2). Thus, the higher scatter visible in Fig. S2 at short times or at the 1 µg/L screen does not propagate to the reported calibration model; at higher concentrations (≥ 50 µg/L) residuals showed no trend and RSDs remained low, so no weighting was required.

#### Derivatization

This study investigated three derivatization approaches. The first approach was a classical derivatization in a vial using a heated stirrer. The other two methods were injector-based derivatizations—one in an SSL and one in a PTV injector.

##### Vial-based derivatization

**Fig. 3:**
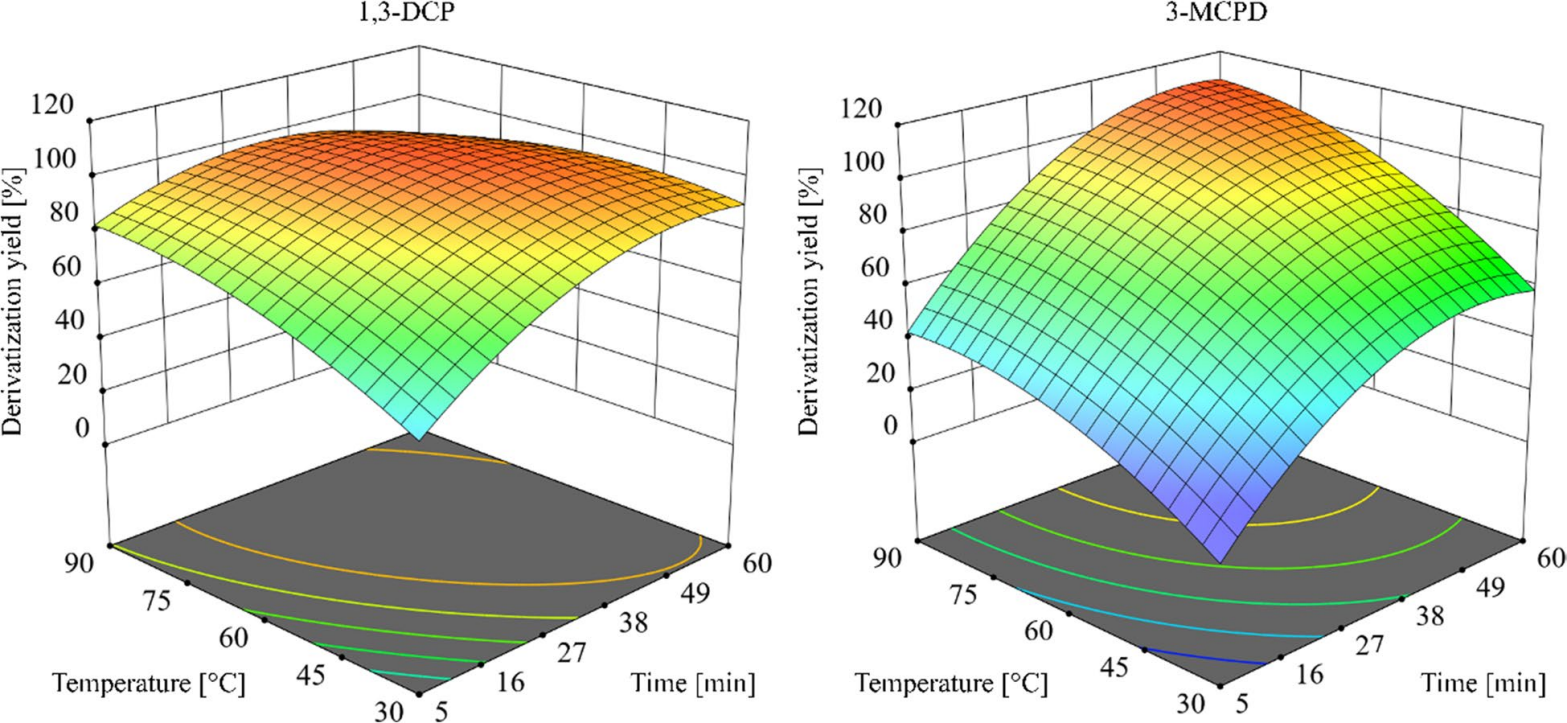
Response surface of the vial-based derivatization, one-factor-at-a-time (OFAT), for 1,3-dichloropropan-2-ol (1,3-DCP) and 3-chloropropane-1,2-diol (3-MCPD). Agitator 450 rpm; analyte 10 µg/L; temperature 30–90 °C in 10 °C steps; aliquots taken every 5 min up to 60 min. Corresponding run-by-run data are listed in Table S4

For vial-based derivatization, a scaled-down MSTFA protocol adapted from Korte et al. was used (10 µL MSTFA; 60 °C; 30 min; 450 rpm) [8]. The resulting 10 µL of MSTFA reduced the consumption of derivatizing reagent by a factor of 5 compared to the reference method [8]. Optimization one-factor-at-a-time (OFAT) scheme: test samples (40 µL of a 1:1 propylene carbonate:ethyl acetate mixture spiked at 10 µg/L with analytes and octafluoronaphthalene (OFN) as internal standard) received 10 µL MSTFA and were incubated in the agitator at 450 rpm. Temperature was stepped in 10 °C increments from 30 to 90 °C; aliquots were sampled every 5 min up to 60 min and analyzed by GC-MS/MS. The details of the individual runs are shown in Table S4. Quadratic surface fitting (Design-Expert v22) yielded *R* ^2^ = 0.71 for 1,3-DCP and *R* ^2^ = 0.92 for 3-MCPD (Fig. 3). Complete conversion in the vial required substantially longer reaction times and higher temperatures for 3-MCPD than for 1,3-DCP; the model indicated a minimum 42 min at ≥ 67 °C to approach full derivatization for both. At the highest temperatures, the response for 1,3-DCP decreased due to evaporation of the TMS derivative from pierced vials (1,3-DCP-TMS boiling point 96 °C)—an observation consistent with derivative volatility and vial-headspace losses [8].

##### SSL injector derivatization

**Fig. 4:**
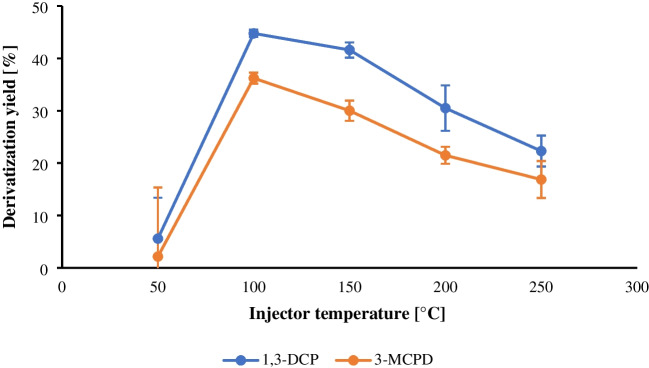
Correlation between SSL injector temperature and derivatization yield (Y_SSL) of analytes 1,3-dichloropropan-2-ol (1,3-DCP) and 3-chloropropane-1,2-diol (3-MCPD) subjected to injector-based derivatization. A sandwich injection protocol was utilized, maintaining a constant analyte concentration of 10 µg/L throughout the experiment. The injector temperature ranged from 50 to 250 °C, with each temperature tested in four replicates. The error bars indicate the relative standard deviation of the fourfold determinations. See Table S6 for a summary of replicate results

 Injector -based derivatization was assessed with a sandwich injection based on the work of M. Nobis [35]: Test samples (20 µL propylene carbonate + 30 µL ethyl acetate, 10 µg/L) were injected with 1 µL MSTFA separated by a 0.2 µL air gap. SSL temperature was varied from 50 °C to 250 °C (four replicates per temperature). To quantify conversion, the derivatization yield in the SSL injector (Y_SSL) was defined as the ratio of the peak area of the derivatized analyte obtained under SSL conditions to that of a fully derivatized vial reference (prepared by heating 20 µL test solution with 20 µL MSTFA at 70 °C for 60 min). The runs and summary of the measurement are shown in Tables S5 and S6. SSL experiments showed a strong dependence on injector temperature (Fig. 4): optimal Y_SSL occurred at 100 °C (45% for 1,3-DCP; 36% for 3-MCPD). At higher temperatures, the MSTFA vaporized too rapidly in the SSL and was lost to the carrier gas before the reaction was complete. At lower injector temperatures, the fixed splitless residence and incomplete vaporization were insufficient to drive derivatization and efficient transfer to the column, which is reflected in the increased variability (higher RSDs).

##### PTV injector derivatization

**Fig. 5:**
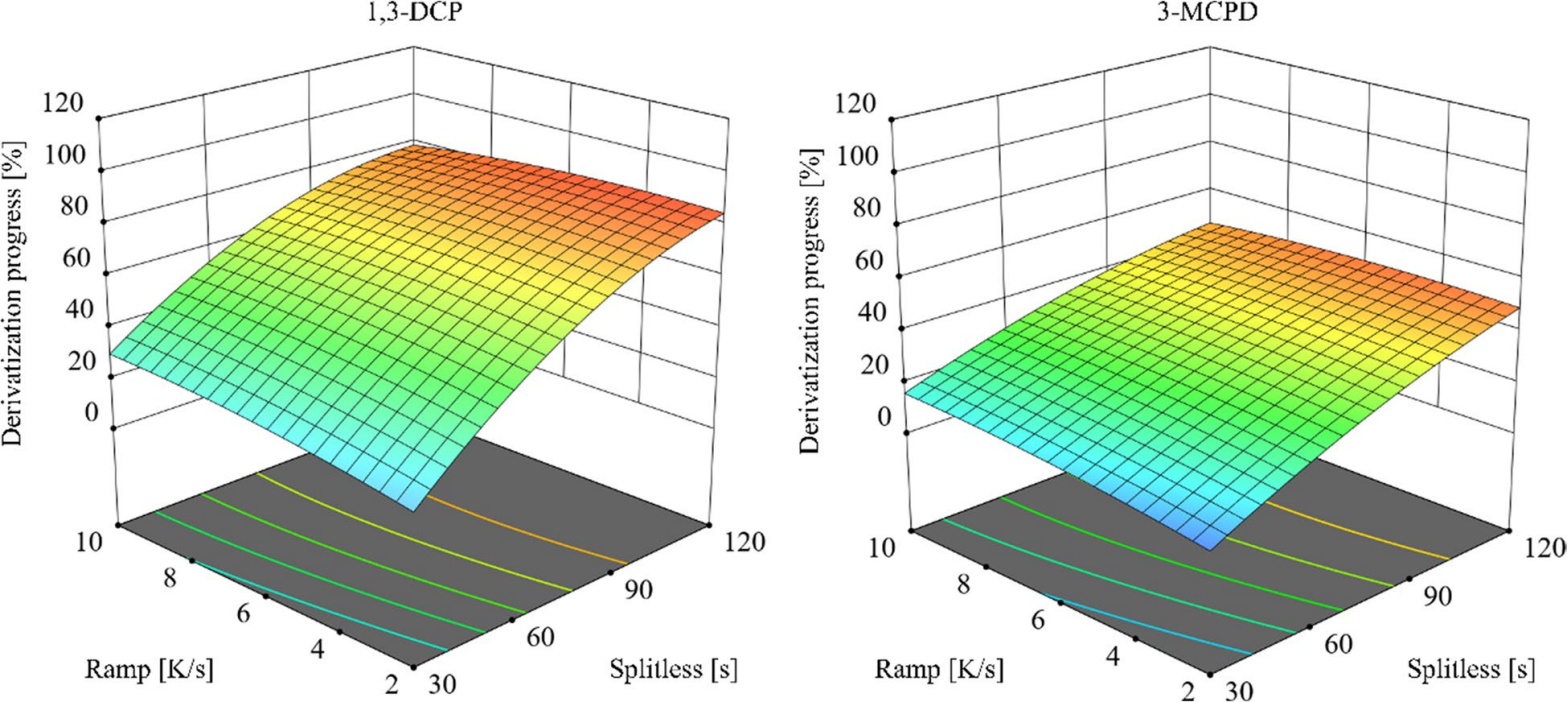
Surface response of the Design of Experiments (DOE) optimization for derivatization of analytes 1,3-dichloropropan-2-ol (1,3-DCP) and 3-chloropropane-1,2-diol (3-MCPD) in-injector derivatization. Derivatization was conducted using a programmed temperature vaporizing injector (PTV injector), with analyte concentration set at 10 µg/L and employing a sandwich injection protocol. The heating rate of the injector (2–10 K/s with a starting temperature of 60 °C and ending at 250 °C injector temperature) and splitless time (30–120 s) were optimized using a central composite design. Corresponding data are listed in Table S6

 Recognizing the critical role of temperature in-injector derivatization, optimization using a PTV injector was carried out using a DOE approach, focusing on two key factors: the injector heating rate and the splitless time [36]. Using the same sandwich injection, analogously Y_PTV was defined as the ratio of the measured peak area to a completely derivatized reference sample. A central composite design (two continuous factors: injector heating rate 2–10 K/s and splitless time 30–120 s; three blocks; five center points per block; total 39 runs) was executed and analyzed with Design-Expert v22 to find the optimal region (Fig. 5). Under the optimized PTV conditions (slow ramp 2 K/s and extended splitless time 120 s), Y_PTV increased substantially relative to Y_SSL: 85% for 1,3-DCP and 49% for 3-MCPD. The fitted quadratic models showed good agreement (*R* ^2^ = 0.96 for 1,3-DCP; *R* ^2^ = 0.93 for 3-MCPD). Full DOE results and run-by-run responses are provided in the supplementary information (Table S7). Despite a derivatization yield of 49% for 3-MCPD, the method was still able to detect analyte concentrations as low as 1 µg/L in the cold water extract to meet compliance testing requirements (Fig. S3).

 The “derivatization yield” reported here is apparent: it captures the net of inlet chemistry and transfer to the column during the splitless window, not the intrinsic chemical conversion alone. In the inlet, MSTFA and analyte coexist briefly in a hot environment; reaction, evaporation, and transport are concurrent [36]. Once MSTFA evaporates, further reaction ceases; once a derivative forms, it is preferentially transported to the column during splitless. Thus, slower heating and longer splitless improve apparent yield by increasing co-residence time of reagent and analyte before venting [36]. Conversely, very high temperatures reduce yield by accelerating MSTFA loss from the liner. The deliberate slowdown in the PTV ramp (2 K/s) is a deliberate design choice that favors reaction before transfer and venting, in contrast to the faster rates seen in other PTV applications [37, 38].

 Potential biases from partial conversion include kinetic competition between the reaction, evaporation and transfer, partial thermal loss or adsorption in the liner and MSTFA depletion due to early evaporation. Since a full mass balance of derivatized and underivatized analytes was not performed, the method employs stable-isotope dilution (d5-1,3-DCP and d5-3-MCPD are added before extraction/derivatization) to correct proportional losses. Validation data, including blind matrix spikes, confirmed the method’s accuracy and precision at the LOQ despite partial conversion (see “Analytical performance characteristics,” Table 2). Carryover was mitigated by opening the split to 100 for 60 s while the injector was hot and then holding it at 10 for the rest of the run. Air blanks injected after 50 µg/L tests showed only baseline noise, confirming negligible memory effects. Future work could investigate mass balance profiling, liner geometry effects and alternative ramp designs (e.g., plateau ramps) to further refine in-injector derivatization.

Considering these trade-offs, the final method selected was injector-based derivatization in the PTV: it provides a faster, more efficient workflow with reduced reagent consumption compared to vial derivatization and delivers acceptable conversion when paired with isotope dilution. If complete derivatization is required, the vial-based approach can be used, accepting the lower sample throughput.

#### Micro-volume sampling module

**Fig. 6:**
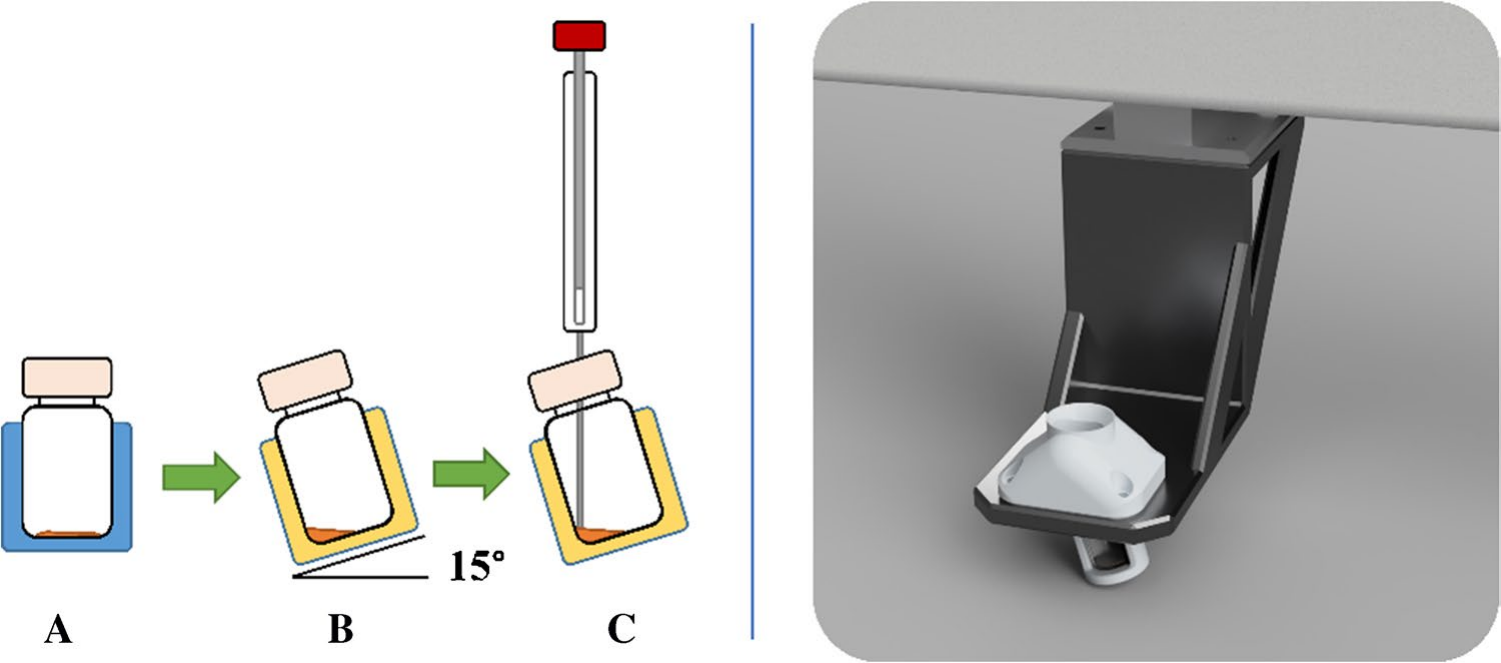
The left part of the image shows a schematic layout of the micro-volume sampling module. A 2-mL vial is transported by the autosampler arm from tray (**A**) to the micro-volume sampling module (**B**) for injection. Unlike the tray design, the micro-volume sampling module has a cavity inclined at a 15° angle. This orientation tilts the vial within the module, causing the liquid to flow towards the lower corner. The autosampler syringe needle is precisely guided by the module to reliably aspirate from small volumes (**C**). The image on the right shows a rendering of the finished micro-volume sampling module attached to an autosampler

To reliably extract small residual volumes (≈50 µL) after evaporation, a simple 15° inclined holder for a standard 2-mL flat-bottom vial was used (CAD in Fusion 360, 3D-printed on a Form 3+; see Fig. 6). The angle concentrates the extract in the lower corner, enabling the autosampler needle to consistently access the liquid; micro-inserts are not necessary. This was a geometric aid, not a new dosing device; the syringe and its specifications remained unchanged.

Micro-insert vials require an additional evaporation/transfer step, increasing effort and potential losses. Vase vials allow aspiration of ~15 µL but proved mechanically unstable during tray transport in the autosampler.

Aspirating 1 µL from ≥ 20 µL (< 5% of the pool) is within the normal capability of a GC autosampler syringe. Because the module does not alter syringe liquid handling, a separate module validation was not performed; suitability was observed during method development. No problems occurred with liquid handling using the module.

The CAD files for the holder are provided openly in the supplementary material to facilitate replication and adaptation (see electronic supplementary material S2). A formal, module-specific validation can be undertaken as future work or by adopting laboratories.

### Analytical performance characteristics

The calibration involved calculating the linear regression equation and the coefficient of determination (*R*^2^) and performing Mandel’s test for linearity for each analyte by spiking the blank matrix with 1,3-DCP and 3-MCPD across 1–150 µg/L. The results showed that both 1,3-DCP and 3-MCPD fit a first-order regression model significantly better than a higher-order model according to the Mandel test. The *R*^2^ values for 1,3-DCP and 3-MCPD were 0.997 and 0.996, respectively, indicating a high degree of linearity.

Repeatability and recovery were determined at 1.0 and 50.0 µg/L by six replicate measurements on the same day and on different days; repeatability is reported as RSD of the six replicates at each level and recovery as the ratio of measured to target concentration. Limits of detection (LOD) and limits of quantification (LOQ) were established according to Eurachem using the blank method [39]: blank matrix extracts with no detectable analyte were processed and measured *n* = 10 times. The results are summarized in Table 2. The VALLME method met the requirements for compliance testing, which specified an LOQ of ≤ 1 µg/L.

**Table 2:** Repeatability (RSD), recovery, and method limits of 1,3-dichloropropan-2-ol (1,3-DCP) and 3-chloropropane-1,2-diol (3-MCPD) in cold water extracts. Limit of detection (LOD) and limit of quantitation (LOQ) were determined according to Eurachem [39]

Analyte	1,3-DCP		3-MCPD	
Intra-day repeatability				
Set concentration (µg/L)	1.0	50.0	1.0	50.0
Mean value (µg/L)	1.0	50.5	0.9	49.7
RSD (%)	3.7	3.6	5.9	3.6
Recovery (%)	102	101	90	99
Inter-day repeatability				
Set concentration (µg/L)	1.0	50.0	1.0	50.0
Mean value (µg/L)	1.0	50.5	0.9	49.7
RSD (%)	3.6	2.6	7.6	4.0
Recovery (%)	102	100	92	99
Method limits				
LOD (µg/L)	0.01		0.16	
LOQ (µg/L)	0.03		0.54	

Uncertainty was estimated using a Eurachem top-down approach [39]. Sixteen replicate measurements of a blind matrix spiked to 1 µg/L, acquired over 6 days, were used to characterize the random component and the bias component. These contributions were combined into a combined standard uncertainty and expanded to ~ 95% coverage with a factor of 2. The resulting mean, recovery, RSD, and expanded uncertainty for both analytes are summarized in Table 3. These values represent uncertainties derived solely from the replicate series; contributions from reference-standard purity, volumetric tolerances, and calibration assignments were not included. Because this work presents a research method rather than an ISO-accredited routine procedure, a full component-resolved ISO 17025 budget and a formal robustness study were out of scope here and are proposed as future work.

**Table 3:** Sixteen replicate determinations of 1,3-dichloropropan-2-ol (1,3-DCP) and 3-chloropropane-1,2-diol (3-MCPD) in a blank cold water extract spiked to 1 µg/L. Summary statistics (mean, recovery, RSD) and the top-down uncertainty estimates (expanded uncertainty, U; *k* = 2) according to Eurachem [39]

Analyte	1,3-DCP	3-MCPD
Mean value (µg/L)	1.0	0.9
RSD (%)	3.4	6.8
Recovery (%)	103	93
Expanded uncertainty U; *k* = 2		
(%)	9.3	21
(µg/L)	± 0.09	± 0.21

External corroboration. An inter laboratory comparison was performed between the automated VALLME workflow (Lab 1) and an independent reference method (Lab 2) on cold water extracts from six samples (two extracts per item); results are summarized in the following chapter.

### Method performance compared to reference method

**Fig. 7:**
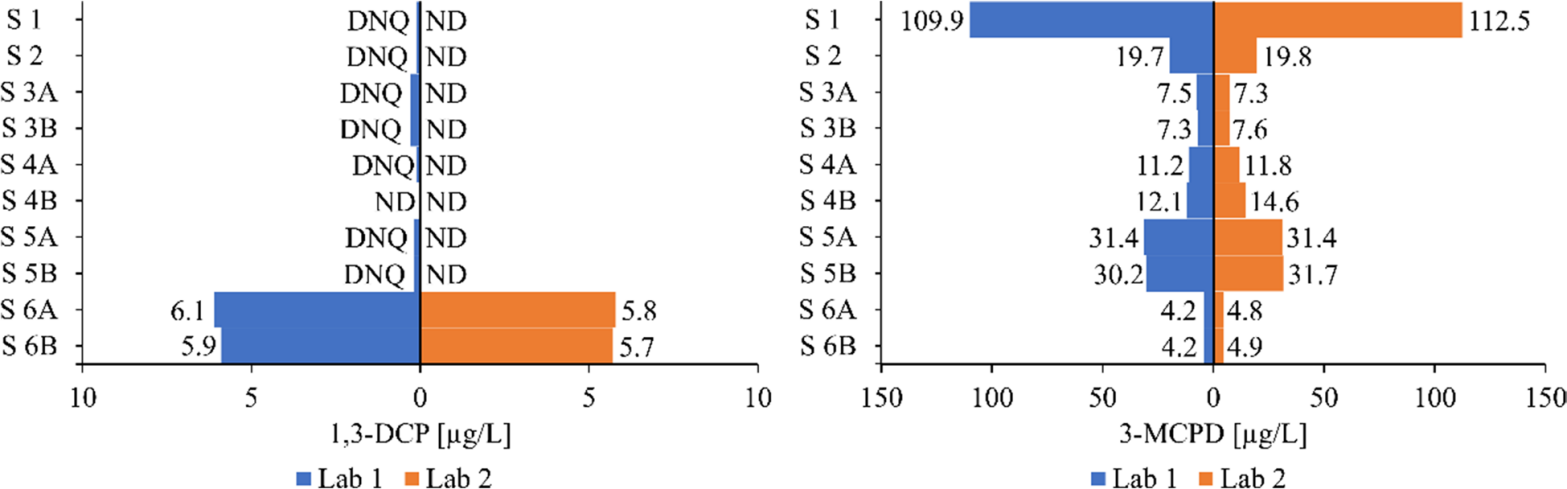
Performance evaluation of the developed VALLME method (Lab 1) versus the reference method (Lab 2) according to Korte et al. (2021) for the analysis of real samples (S) [8]. Samples A and B are two different CWEs from the same sample. DNQ, detected, not quantifiable; ND, not detected

The performance of the developed VALLME method (Lab 1) and the reference method (Lab 2) for the analysis of real samples was evaluated by comparing the results obtained from cold water extracts of six different samples. Samples 3 to 6 included two cold water extracts (Sample A and Sample B) prepared from the same paper article, respectively. The developed method showed lower LODs of 0.01 µg/L and 0.16 µg/L for 1,3-DCP and 3-MCPD, respectively, while the reference method had LODs of 0.4 µg/L for both analytes [8]. The results of the measurements are shown in Fig. 7. The dataset is available in supplementary material (electronic supplementary material S3). The developed method was able to detect 1,3-DCP in all samples except Sample 4B, while the reference method reported non detectable (ND) values for the first 5 samples. The results for 3-MCPD were comparable between the two methods, with no significant differences observed. The developed method demonstrated good precision and comparable results to the reference method when analyzing real samples contaminated with 1,3-DCP and 3-MCPD.

### Greenness evaluation

**Fig. 8:**
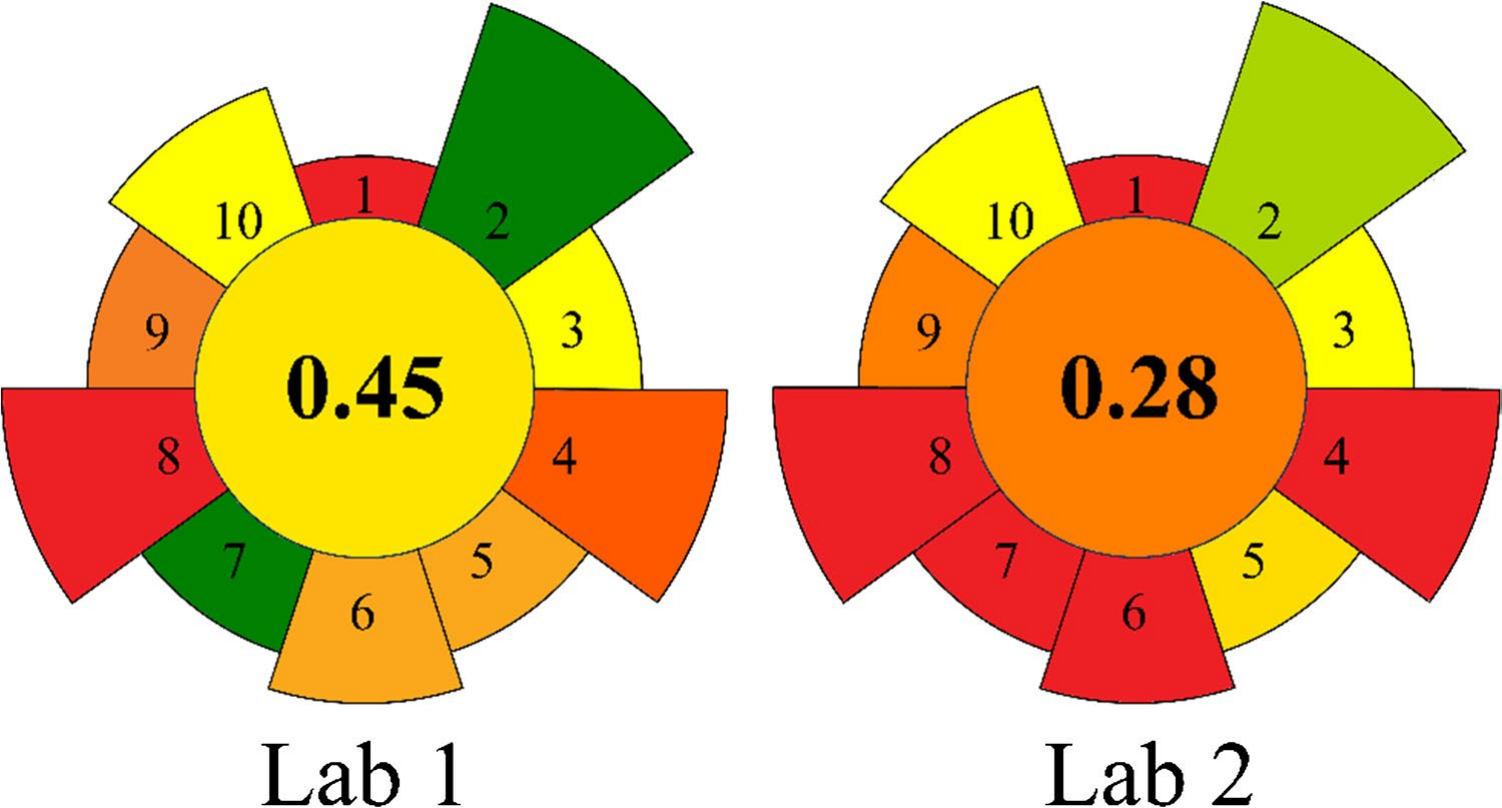
Evaluation of the sustainability of the developed VALLME method compared to the reference method using the AGREEprep concept. The developed VALLME method (Lab 1) scores higher (0.45) than the reference method (Lab 2) due to reduced reagent consumption, less waste generation, full automation, and increased sample throughput. Both methods have high energy consumption due to GC-MS(/MS) systems, which is essential for analysis in the lower µg/L range

To assess the comparative environmental friendliness and sustainability of the developed VALLME method against the reference method, the AGREEprep concept was employed (Fig. 8). This concept evaluates 10 distinct method parameters, assigning ratings between 0 and 1 with varied weightings. The parameters encompass aspects such as material and chemical sustainability and reusability, sample throughput, degree of automation, and energy consumption per sample. The standard weighting scheme for these parameters was upheld in the comparison between the VALLME method developed in this work and the reference method [7, 8]. The VALLME method (Lab 1) outperforms the reference method (Lab 2) with a score of 0.45 compared to 0.28. This stems from several factors: lower consumption of the extraction solvent (1.2 mL per analysis compared to 70 mL), reduced utilization of derivatization reagent (1 µL per analysis compared to 50 µL), resulting in reduced use of hazardous materials (parameter 2); generation of less waste per analysis (approximately 17 g versus 93 g for the reference method, parameter 4); complete automation of sample preparation (parameter 6); and higher sample throughput (4 vs. 1.2 samples/h) (parameter 7). While both methods exhibit high energy consumption due to the utilization of GC-MS(/MS) measurement systems (parameters 8 and 9), this is inevitable given the need for analysis in the lower µg/L range.

AGREEprep evaluates resource use, safety, and operational features; it does not encode analytical conversion (derivatization efficiency) or extraction yield. Analytical performance is therefore treated separately: despite partial in-inlet conversion (PTV) and low raw extraction for 3-MCPD in single-step VALLME, stable-isotope dilution (labels added pre-extraction/derivatization) delivered the required LOQ and precision (Table 2). Where a laboratory’s quality policy requires higher uncorrected extraction or conversion, the workflow can be adapted (e.g., multistep VALLME, DLLME, or µSPE and vial-based derivatization). Such adaptations generally increase reagents, consumables, and waste and would lower the AGREEprep score correspondingly (principally criteria 2, 4 and 7). Table S8 lists all AGREEprep inputs and per-criterion scores.

## Conclusion

A lean, fully automated VALLME-GC-MS/MS workflow with in-inlet (PTV) derivatization for 1,3-DCP and 3-MCPD in cold water extracts of paper and board was developed. The workflow reduces derivatization reagent consumption by ~50-fold and lowers extraction solvent use from 70 to 1.2 mL per analysis, while maintaining robust analytical performance. Automation decreased manual steps (2 vs. 5) and increased throughput (≈4 vs. 1.2 samples/h). Matrix-matched calibration (1–150 µg/L) was linear by Mandel’s test and method LOQs (0.03 µg/L for 1,3-DCP; 0.54 µg/L for 3-MCPD) meet compliance needs. Despite partial in-inlet conversion (≈85% and ≈49% apparent yields in PTV for 1,3-DCP and 3-MCPD, respectively), isotope-dilution with labels added pre-extraction/derivatization enabled accurate quantification, as confirmed by validation in a blind matrix and a top-down uncertainty estimate at 1 µg/L.

The reported “derivatization yield” represents the net effect of reaction and transfer in the inlet; future studies may quantify mass balance (derivatized plus underivatized species), assess liner geometry/surface effects, and explore alternative heating profiles (e.g., ramp plateaus) to further improve in-inlet conversion—particularly for 3-MCPD. Single-step VALLME retains a low raw extraction for 3-MCPD; where higher uncorrected recovery or complete derivatization is a formal requirement, the workflow can be adapted (multistep VALLME, DLLME, or µSPE and vial-based derivatization), acknowledging trade-offs in consumables, throughput, and greenness. A full ISO-17025 component-resolved uncertainty budget and a structured robustness and ruggedness screen (e.g., Youden or fractional-factorial variation of vortex speed, extraction time, injection volume and PTV settings) are appropriate targets for subsequent work or adoption in routine environments. To facilitate replication and further development, AGREEprep inputs, instrument methods, and CAD files for the micro-volume sampling module are provided in the supplementary information.

Overall, the presented workflow offers a practical balance of automation, analytical reliability, and sustainability for routine surveillance of chloropropanols in paper-based food contact materials, with clear avenues for performance enhancement where required by local quality policies.

## Supplementary Information

Below is the link to the electronic supplementary material.

ESM 1 (DOCX 80.2 KB)

ESM 2 (DOCX 12.5 KB)

ESM 3 (DOCX 322 KB)

ESM 4 (DOCX 1.99 MB)

## Data Availability

Instrument method files for the autosampler, PTV, and GC-MS/MS are provided as ESM_1. CAD models (STEP) for the micro-volume sampling module are provided as ESM_2. The dataset underpinning the method-comparison study (Lab 1 vs. Lab 2) is provided as ESM_3. Due to the file size, additional raw files are available upon request from the corresponding author.
