# Supplementary material for: Automated vortex-assisted-liquid-liquid microextraction with injector-based derivatization for GC-MS/MS analysis of 1,3-dichloropropan-2-ol and 3-chloropropane-1,2-diol in food contact paper products
Source: Anal Bioanal Chem. 2025 Oct 21;417(28):6413–25. doi: 10.1007/s00216-025-06133-2 (PMC12596282; doi:10.1007/s00216-025-06133-2)
Supplement: Supplementary file 1 — ESM 1 (DOCX 80.2 KB) [file 216_2025_6133_MOESM1_ESM.docx]

# Supplementary Information

**Automated vortex-assisted-liquid-liquid microextraction with injector-based derivatization for GC-MS/MS analysis of 1,3-dichloropropan-2-ol and 3-chloropropane-1,2-diol in food contact paper products**

**Malte Hübschen^a,b^, Fabrian Brenz^c^, Torsten C. Schmidt^a*^**

^a^ Instrumental Analytical Chemistry, University of Duisburg-Essen, Universitätsstraße 5, 45141, Essen, Germany
^b^ Axel Semrau GmbH, Stefansbecke 42, 45549 Sprockhövel, Germany
^c^ CVUA-MEL, Joseph-König-Straße 40, 48147 Münster, Germany

^*^ Corresponding author:
Torsten C. Schmidt, e-mail: torsten.schmidt@uni-due.de, address: Faculty of Chemistry, Instrumental Analytical Chemistry, University of Duisburg-Essen, Universitätsstraße 5, 45141 Essen, Germany

ORCID ID:
Torsten C. Schmidt: 0000-0003-1107-4403

Table S1 The results of optimizing the extraction solvent for the analytes 1,3-dichloro-2-propanol (1,3-DCP) and 3-chloropropane-1,2-diol (3-MCPD) in the solvents methyl tert-butyl ether (MTBE), diethyl ether (ET2O), and ethyl acetate (EtOAc) were matched. The table shows the recovery that was experimentally determined (Exp. rec.) with the respective relative standard deviation (RSD), compared with the recovery calculated from the UFZ-LSER database (Cal. rec.). Those from the UFZ-LSER database refer to a water-solvent mixture.

| Extraction agent | 1,3-DPC | | | 3-MCPD | | |
| --- | --- | --- | --- | --- | --- | --- |
|  | Exp. rec. [%] | RSD  [%] | calc.  Rec [%] | Exp. rec. [%] | RSD  [%] | calc.  Rec [%] |
| MTBE | 84 | 1.2 | 20 | 4.2 | 3.9 | 4.3 |
| Et₂O | 87 | 1.7 | 23 | 2.8 | 4.8 | 2.8 |
| EtOAc | 94 | 1.9 | 25 | 9.0 | 4.8 | 9.1 |

Table S2 Comparison of the logarithmic partition coefficients (log*K*) determined in the experiment (Exp.) with those of the UFZ-LSER database for the analytes 1,3-dichloro-2-propanol (1,3-DCP) and 3-chloropropane-1,2-diol (3-MCPD) in the solvents methyl tert-butyl ether (MTBE), diethyl ether (ET2O) and ethyl acetate (EtOAc). The exp. log*K* refer to water and solvent mixture saturated with sodium chloride. Those of the UFZ-LSER database refer to a water-solvent mixture.

| Extraction solvent | Exp.  log*K* 1,3-DPC | LSER  log*K* 1,3-DPC | Exp.  log*K* 3-MCPD | LSER  log*K* 3-MCPD |
| --- | --- | --- | --- | --- |
| MTBE | 1.63 | 0.32 | -0.43 | -0.93 |
| Et₂O | 1.76 | 0.39 | -0.62 | -0.83 |
| EtOAc | 2.09 | 0.45 | -0.08 | -0.73 |

Table S3 Results of optimizing the vortex extraction time for the analytes 1,3-dichloro-2-propanol (1,3-DCP) and 3-chloropropane-1,2-diol (3-MCPD) at concentrations of 1 and 50 µg/L using ethyl acetate (EtOAc) as the extraction solvent. The experimentally determined recoveries (rec.) with the respective relative standard deviations (RSD) are shown.

| Vortex  extraction  time [min] | 1.3-DCP  (1 µg/L) | | 1.3-DCP  (50 µg/L) | | 3-MCPD  (50 µg/L) | | 3-MCPD  (50 µg/L) | |
| --- | --- | --- | --- | --- | --- | --- | --- | --- |
|  | Rec. [%] | RSD [%] | Rec. [%] | RSD [%] | Rec. [%] | RSD [%] | Rec. [%] | RSD [%] |
| 1 | 113 | 25 | 97 | 2.4 | 97 | 47 | 96 | 8.8 |
| 2 | 77 | 17 | 99 | 3.0 | 100 | 34 | 97 | 5.1 |
| 3 | 101 | 27 | 101 | 3.4 | 101 | 27 | 100 | 4.8 |
| 5 | 103 | 8.0 | 101 | 4.6 | 83 | 30 | 101 | 3.4 |
| 7 | 97 | 13 | 102 | 2.8 | 117 | 27 | 104 | 2.5 |
| 10 | 110 | 15 | 100 | 1.7 | 104 | 33 | 102 | 2.9 |

**Fig S1** Effect of vortex time on raw extraction yield in VALLME at 2000 rpm in blank cold-water extract. All determinations were performed five times at different time intervals and two different concentration levels for 1,3-dichloro-2-propanol (1,3-DCP) and 3‑chloropropane-1,2-diol (3-MCPD). Internal standards were added post-extraction in this experiment, so values represent raw extraction yields with no isotope correction. Error bars denote the RSD of the fivefold determination

Table S4 One-factor-at-a-time (OFAT) scheme dataset for vial-based derivatization of 1,3-dichloropropan-2-ol (1,3 DCP) and 3‑chloropropane-1,2-diol (3-MCPD) at 10 µg/L. 84 runs comprising 7 temperatures (30–90 °C in 10 °C steps) and 12 time points (5–60 min in 5-min steps). Agitator 450 rpm; MSTFA 10 µL; test solution 40 µL (1:1 propylene carbonate:ethyl acetate).

| Run | Time  [min] | Temperature [°C] | Derivatization yield [%] | |
| --- | --- | --- | --- | --- |
|  |  |  | 1,3-DCP | 3-MCPD |
| 1 | 5 | 30 | 33 | 19 |
| 2 | 10 | 30 | 58 | 27 |
| 3 | 15 | 30 | 70 | 32 |
| 4 | 20 | 30 | 74 | 40 |
| 5 | 25 | 30 | 81 | 44 |
| 6 | 30 | 30 | 91 | 40 |
| 7 | 35 | 30 | 82 | 46 |
| 8 | 40 | 30 | 89 | 54 |
| 9 | 45 | 30 | 91 | 58 |
| 10 | 50 | 30 | 95 | 62 |
| 11 | 55 | 30 | 92 | 64 |
| 12 | 60 | 30 | 90 | 67 |
| 13 | 5 | 40 | 42 | 21 |
| 14 | 10 | 40 | 58 | 31 |
| 15 | 15 | 40 | 81 | 38 |
| 16 | 20 | 40 | 81 | 48 |
| 17 | 25 | 40 | 92 | 53 |
| 18 | 30 | 40 | 86 | 52 |
| 19 | 35 | 40 | 92 | 56 |
| 20 | 40 | 40 | 86 | 66 |
| 21 | 45 | 40 | 94 | 66 |
| 22 | 50 | 40 | 91 | 67 |
| 23 | 55 | 40 | 96 | 71 |
| 24 | 60 | 40 | 89 | 69 |
| 25 | 5 | 50 | 60 | 32 |
| 26 | 10 | 50 | 74 | 36 |
| 27 | 15 | 50 | 94 | 55 |
| 28 | 20 | 50 | 91 | 57 |
| 29 | 25 | 50 | 90 | 62 |
| 30 | 30 | 50 | 89 | 65 |
| 31 | 35 | 50 | 94 | 79 |
| 32 | 40 | 50 | 96 | 87 |
| 33 | 45 | 50 | 102 | 91 |
| 34 | 50 | 50 | 104 | 91 |
| 35 | 55 | 50 | 99 | 101 |
| 36 | 60 | 50 | 92 | 101 |
| 37 | 5 | 60 | 68 | 30 |
| 38 | 10 | 60 | 91 | 42 |
| 39 | 15 | 60 | 93 | 57 |
| 40 | 20 | 60 | 105 | 71 |
| 41 | 25 | 60 | 103 | 83 |
| 42 | 30 | 60 | 100 | 87 |
| 43 | 35 | 60 | 93 | 94 |
| 44 | 40 | 60 | 94 | 101 |
| 45 | 45 | 60 | 95 | 97 |
| 46 | 50 | 60 | 97 | 103 |
| 47 | 55 | 60 | 97 | 102 |
| 48 | 60 | 60 | 85 | 94 |
| 49 | 5 | 70 | 67 | 28 |
| 50 | 10 | 70 | 89 | 45 |
| 51 | 15 | 70 | 102 | 60 |
| 52 | 20 | 70 | 98 | 75 |
| 53 | 25 | 70 | 90 | 75 |
| 54 | 30 | 70 | 96 | 88 |
| 55 | 35 | 70 | 99 | 92 |
| 56 | 40 | 70 | 91 | 91 |
| 57 | 45 | 70 | 95 | 98 |
| 58 | 50 | 70 | 100 | 108 |
| 59 | 55 | 70 | 93 | 99 |
| 60 | 60 | 70 | 97 | 104 |
| 61 | 5 | 80 | 74 | 31 |
| 62 | 10 | 80 | 95 | 52 |
| 63 | 15 | 80 | 89 | 60 |
| 64 | 20 | 80 | 93 | 89 |
| 65 | 25 | 80 | 97 | 98 |
| 66 | 30 | 80 | 100 | 97 |
| 67 | 35 | 80 | 86 | 102 |
| 68 | 40 | 80 | 98 | 110 |
| 69 | 45 | 80 | 98 | 105 |
| 70 | 50 | 80 | 104 | 115 |
| 71 | 55 | 80 | 94 | 103 |
| 72 | 60 | 80 | 96 | 105 |
| 73 | 5 | 90 | 82 | 34 |
| 74 | 10 | 90 | 98 | 55 |
| 75 | 15 | 90 | 91 | 75 |
| 76 | 20 | 90 | 103 | 98 |
| 77 | 25 | 90 | 84 | 92 |
| 78 | 30 | 90 | 95 | 110 |
| 79 | 35 | 90 | 84 | 95 |
| 80 | 40 | 90 | 89 | 101 |
| 81 | 45 | 90 | 98 | 100 |
| 82 | 50 | 90 | 87 | 98 |
| 83 | 55 | 90 | 91 | 98 |
| 84 | 60 | 90 | 98 | 101 |

Table S5 Run-by-run dataset for the split/splitless (SSL) injector temperature study. 1,3-dichloropropan-2-ol (1,3 DCP) and 3‑chloropropane-1,2-diol (3-MCPD) under sandwich injection at 10 µg/L. SSL derivatization yield (Y_SSL) for 1,3-DCP and 3‑MCPD at 50-250 °C with four replicates per temperature (n = 4).

| Run | Temperatur [°C] | Y_SSL [%] | |
| --- | --- | --- | --- |
|  |  | 1,3-DCP | 3-MCPD |
| 1 | 50 | 6.2 | 2.6 |
| 2 | 50 | 5.8 | 2.2 |
| 3 | 50 | 5.0 | 1.8 |
| 4 | 50 | 5.4 | 2.0 |
| 5 | 75 | 44 | 37 |
| 6 | 75 | 45 | 36 |
| 7 | 75 | 45 | 37 |
| 8 | 75 | 45 | 36 |
| 9 | 100 | 42 | 30 |
| 10 | 100 | 42 | 29 |
| 11 | 100 | 42 | 31 |
| 12 | 100 | 41 | 30 |
| 13 | 150 | 32 | 22 |
| 14 | 150 | 31 | 21 |
| 15 | 150 | 31 | 22 |
| 16 | 150 | 28 | 21 |
| 17 | 200 | 23 | 17 |
| 18 | 200 | 23 | 17 |
| 19 | 200 | 23 | 17 |
| 20 | 200 | 21 | 16 |

Table S6 Summary of SSL injector temperature vs derivatization yield (Y_SSL) for 1,3-dichloropropan-2-ol (1,3 DCP) and 3‑chloropropane-1,2-diol (3-MCPD) under sandwich injection at 10 µg/L. Temperatures 50-250 °C were tested in four replicates per point. Y_SSL is reported as % relative to a fully derivatized vial reference; RSD (%) denotes the relative standard deviation across replicates

| SSL injector  temperature [°C] | 1,3-DCP | | 3-MCPD | |
| --- | --- | --- | --- | --- |
|  | Y_SSL [%] | RSD [%] | Y_SSL [%] | RSD [%] |
| 50 | 5.6 | 7.82 | 2.9 | 13.19 |
| 100 | 45 | 0.68 | 36 | 1.06 |
| 150 | 42 | 1.45 | 30 | 1.93 |
| 200 | 30 | 4.33 | 21 | 1.62 |
| 250 | 22 | 2.95 | 17 | 3.55 |

Table S7 Central composite design (CCD) dataset for programmed temperature vaporizing injector (PTV injector) derivatization yield (Y_PTV) optimization of 1,3-dichloropropan-2-ol (1,3 DCP) and 3-chloropropane-1,2-diol (3-MCPD) at 10 µg/L under sandwich injection. Factors: splitless time (30-120 s; axial points at 11.4 and 138.6 s) and heating ramp (2-10 K/s; axial points at 0.3 and 11.7 K/s); inlet from 60 °C to 250 °C. Three blocks with five center points per block; 39 runs in total. Y_PTV is expressed relative to a fully derivatized vial reference.

| Run | Blocks |  | Splitless time [s] | Ramp [K/s] | Y_PTV [%] | |
| --- | --- | --- | --- | --- | --- | --- |
|  |  |  |  |  | 1,3-DCP | 3-MCPD |
| 1 | Block 1 |  | 11.4 | 6 | 3 | 1 |
| 2 | Block 1 |  | 120 | 2 | 88 | 53 |
| 3 | Block 1 |  | 75 | 6 | 64 | 34 |
| 4 | Block 1 |  | 75 | 6 | 66 | 35 |
| 5 | Block 1 |  | 30 | 10 | 26 | 12 |
| 6 | Block 1 |  | 75 | 0.3 | 54 | 23 |
| 7 | Block 1 |  | 30 | 2 | 22 | 9 |
| 8 | Block 1 |  | 75 | 6 | 65 | 35 |
| 9 | Block 1 |  | 75 | 11.7 | 67 | 37 |
| 10 | Block 1 |  | 75 | 6 | 69 | 37 |
| 11 | Block 1 |  | 75 | 6 | 70 | 40 |
| 12 | Block 1 |  | 138.6 | 6 | 86 | 52 |
| 13 | Block 1 |  | 120 | 10 | 77 | 46 |
| 14 | Block 2 |  | 30 | 10 | 27 | 13 |
| 15 | Block 2 |  | 75 | 0.3 | 54 | 24 |
| 16 | Block 2 |  | 75 | 6 | 64 | 32 |
| 17 | Block 2 |  | 75 | 6 | 64 | 33 |
| 18 | Block 2 |  | 75 | 6 | 72 | 40 |
| 19 | Block 2 |  | 30 | 2 | 19 | 8 |
| 20 | Block 2 |  | 75 | 6 | 78 | 43 |
| 21 | Block 2 |  | 120 | 10 | 77 | 45 |
| 22 | Block 2 |  | 75 | 6 | 61 | 31 |
| 23 | Block 2 |  | 138.6 | 6 | 83 | 51 |
| 24 | Block 2 |  | 11.4 | 6 | 4 | 1 |
| 25 | Block 2 |  | 120 | 2 | 88 | 54 |
| 26 | Block 2 |  | 75 | 11.7 | 68 | 39 |
| 27 | Block 3 |  | 11.4 | 6 | 4 | 1 |
| 28 | Block 3 |  | 75 | 6 | 58 | 29 |
| 29 | Block 3 |  | 120 | 2 | 89 | 54 |
| 30 | Block 3 |  | 75 | 0.3 | 55 | 25 |
| 31 | Block 3 |  | 30 | 2 | 18 | 7 |
| 32 | Block 3 |  | 75 | 11.7 | 68 | 37 |
| 33 | Block 3 |  | 75 | 6 | 66 | 36 |
| 34 | Block 3 |  | 75 | 6 | 66 | 36 |
| 35 | Block 3 |  | 120 | 10 | 76 | 44 |
| 36 | Block 3 |  | 75 | 6 | 67 | 37 |
| 37 | Block 3 |  | 75 | 6 | 62 | 31 |
| 38 | Block 3 |  | 138.6 | 6 | 83 | 51 |
| 39 | Block 3 |  | 30 | 10 | 26 | 13 |


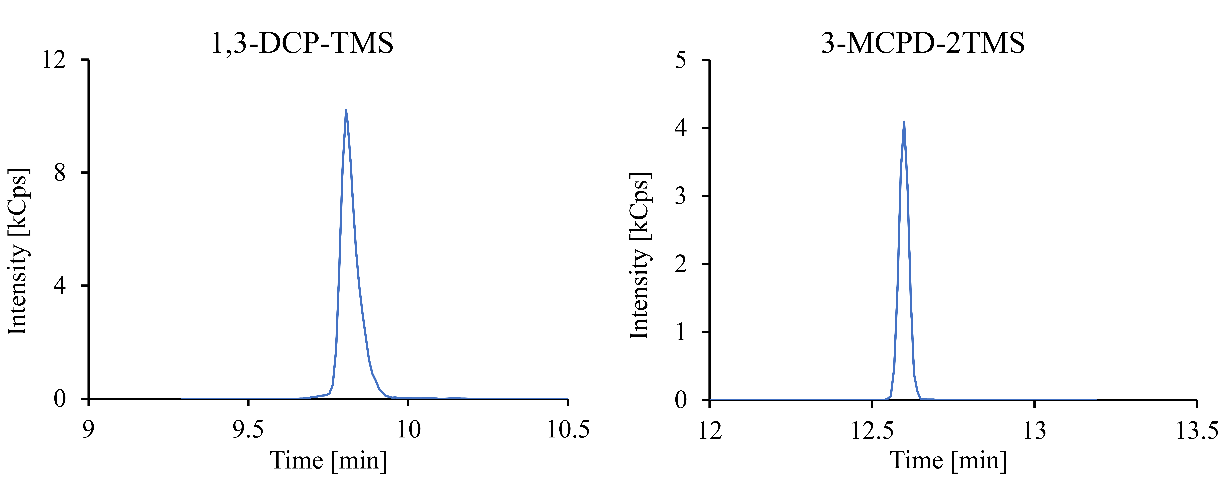


**Fig S2** GC-MS/MS chromatograms of trimethylsilyl (TMS) derivatives of 1,3-dichloro-2-propanol (1,3-DCP) and 3‑chloropropane-1,2-diol (3-MCPD) from an automatically processed blank cold-water extract spiked to 1 µg/L. 1,3-DCP-TMS has a retention time of 9.8 min (signal height 10.2 kCps), and 3-MCPD-2TMS elutes at 12.6 min (signal height 4.1 kCps)

Table S8 AGREEprep input values for the greenness assessment of sample preparation. Criteria, brief description, weights, values and score for the VALLME method and the reference method.

| Criterion | Criterion description | Weights | VALLME method | | Reference method | |
| --- | --- | --- | --- | --- | --- | --- |
|  |  |  | Value | Score | Value | Score |
| 1 | Sample prep. placement | 1 | Ex situ | 0.00 |  | 0.00 |
| 2 | Hazardous materials [g] | 5 | 0.01 | 1.00 | 0.1 | 0.67 |
| 3 | Sustainability | 2 | 50-75%, used ONCE | 0.50 | 50-75%, used ONCE | 0.50 |
| 4 | Waste [g] | 4 | 17 | 0.17 | 93 | 0.00 |
| 5 | Sample size [mL] | 2 | 10 | 0.33 | 5 | 0.42 |
| 6 | Throughput [samples/h] | 3 | 4 | 0.33 | 2 | 0.04 |
| 7 | Integration and automation | 2 | 2 steps; fully automated | 1.00 | 5 steps; manual systems | 0.06 |
| 8 | Energy consumption [W/sample] | 4 | 360 | 0.08 | 306 | 0.12 |
| 9 | Analysis technique | 2 | GC-MS/MS | 0.25 | GC-MS | 0.25 |
| 10 | Operator's safety | 3 | 2 hazards | 0.50 | 2 hazards | 0.50 |
